# Relationships between adiponectin and matrix metalloproteinase-9 (MMP-9) serum levels and postoperative cognitive dysfunction in elderly patients after general anesthesia

**DOI:** 10.1007/s40520-015-0519-9

**Published:** 2016-01-14

**Authors:** Haihui Xie, Dehui Huang, Shu Zhang, Xiaoming Hu, Jianer Guo, Zaiguo Wang, Guilan Zhou

**Affiliations:** 1 Department of Anesthesiology, Dongguan People’s Hospital, Dongguan, 5230181 Guangdong China; 2 Department of Osteology, Dongguan People’s Hospital, Dongguan, 5230181 Guangdong China

**Keywords:** Postoperative cognitive dysfunction, Adiponectin, Matrix metalloproteinase-9, The elderly, General anesthesia

## Abstract

**Objective:**

The aim of this study was to evaluate the relationships between the serum levels of adiponectin (ADP) and matrix metalloproteinase-9 (MMP-9) and postoperative cognitive dysfunction (POCD) in elderly patients after general anesthesia.

**Methods:**

The cognitive functions of 98 elderly patients who were scheduled to undergo selective hip replacement surgery under general anesthesia were assessed using the Montreal Cognitive Assessment (MoCA) 3 days before surgery and on postoperative Days 1, 2, 3, and 7. The serum levels of ADP and MMP-9 were determined at the same time points, and the presence of POCD on postoperative Day 3 was recorded. The patients were divided into a POCD group and non-POCD group.

**Results:**

Postoperative cognitive dysfunction was observed in 28 patients (28.5 %). Serum MMP-9 levels significantly increased and serum ADP levels significantly decreased in the POCD group at each postoperative time point and in the non-POCD group on postoperative Days 1 and 2 compared to the presurgical levels. Serum MMP-9 levels were significantly higher and serum ADP levels were significantly lower in the POCD group compared with those in the non-POCD group at each time point. In the POCD patients, serum MMP-9 levels were significantly and negatively correlated and serum ADP levels were significantly and positively correlated with the MoCA scores.

**Conclusions:**

The increased serum MMP-9 levels and decreased serum ADP levels in elderly patients after general anesthesia might be involved in the POCD pathophysiological process.

## Introduction

Postoperative cognitive dysfunction (POCD), which is a common postoperative complication in elderly patients, delays patient recovery and prolongs their hospitalization. The occurrence of POCD might be related to multiple risk factors, including anesthesia, surgery, and perioperative status, and the mechanisms underlying its occurrence are currently unclear. Adiponectin (ADP), which is a protein hormone that is derived from fat cells and is secreted by fat tissues, has many bioactivities, including anti-inflammation, anti-oxidation, and blood sugar reduction. Thus, ADP might have protective effects towards cells, tissues, and organs [1–5], and ADP has been shown to effectively reduce the secretion of central proinflammatory cytokines [6]. The main functions of matrix metalloproteinase-9 (MMP-9), which is a member of a group of Zn^2+^-dependent proteolytic enzymes, are to degrade and remodel the extracellular matrix. MMP-9 plays a major role in degrading the basal membrane. The abnormal expression of MMP-9 has been directly related to disruption of the blood–brain barrier, and it is a key factor in vasogenic edema and secondary brain injuries. The overexpression of MMP-9 affects the integrity of the vascular basal membrane, thus resulting in severe blood–brain barrier damage and the entry of plasma proteins and a variety of substances and metabolites into the brain, which causes vasogenic edema and the entry of inflammatory cells into brain tissues [7–9]. Reductions in the expression of MMP-9 have been shown to significantly improve cerebral ischemia–reperfusion injuries in rats [10]. However, it is unclear if serum ADP and MMP-9 are involved in the pathophysiological processes underlying POCD. This study was designed to investigate the relationships between serum ADP and MMP-9 levels and POCD in elderly patients after general anesthesia in order to better understand the prevention and treatment of POCD.

## Materials and methods

### Case selection

This study was conducted in accordance with the Declaration of Helsinki. This study was conducted with approval from the Ethics Committee of Dongguan People’s Hospital. Written informed consent was obtained from all participants. A total of 98 elderly patients who were scheduled to undergo selective total hip replacement surgery while under general anesthesia were enrolled in the study. The patients were classified as grades II or III of the American Society of Anesthesiologists. They had an age range of 65–83 years and included 63 males and 35 females. The exclusion criteria were any of the following: (1) Montreal Cognitive Assessment (MoCA) score less than 26 points 3 days before the surgery; (2) inability to recognize the text on the cognitive test forms; (3) comorbidity of significant diseases of the respiratory system, circulatory system, liver, kidney, or nervous system; (4) presence of diabetes before the surgery; (5) treatment with antipsychotic drugs, sedatives, or narcotic analgesics within 2 years; (6) history of alcohol abuse or drug dependence; (7) comorbidity of severe visual or hearing impairments or other reasons that would cause communication problems with the interviewer; (8) unsatisfactory surgical results necessitating an immediate second surgery; (9) operation time over 3 h; or (10) intraoperative blood loss over 800 mL. The patients were divided into a POCD group and non-POCD group based on the presence of POCD 3 days after the surgery.

### Anesthetic methods

The patients were fasted for 8–10 h and forbidden water for 4 h before the surgery. After entering the operating room and confirming the patient, the peripheral vein of the upper extremity was opened, and radial artery puncture was performed. Electrocardiograms, peripheral capillary oxygen saturation, respiratory rate, and partial pressure of end-tidal carbon dioxide were monitored, and a Narcotrend monitor was used to measure the depth of anesthesia. All of the patients were treated with endotracheal general anesthesia without preoperative medication. General anesthesia was induced by intravenous injections of midazolam (0.05–0.1 mg/kg, Jiangsu Nhwa Pharmaceutical Corporation Ltd., Xuzhou, China), etomidate (0.3 mg/kg, Jiangsu Nhwa Pharmaceutical Corporation Ltd.), fentanyl (3–5 μg/kg, Yichang Humanwell Pharmaceutical Co., Ltd., Yichang, China), and cisatracurium [0.2 mg/kg, Dong Ying (Jiangsu) Pharmaceutical Co., Ltd., Nantong City, China]. Mechanical ventilation was performed after intubation, and the partial pressure of the end-tidal carbon dioxide was maintained at 35–45 mmHg. Intraoperative anesthesia was maintained by a constant infusion of propofol (Xi’an Libang Pharmaceutical Co., Ltd., Xi’an, China), remifentanil (Yichang Humanwell Pharmaceutical Co., Ltd.), and cisatracurium, and the Narcotrend index was maintained at D2-E1. During the surgery, urine output, blood loss, and transfusion volume were recorded, and the hemodynamics were kept stable. Patient-controlled interscalene analgesia was administered postoperatively. The analgesics (10 μg/kg fentanyl, 200 mg flurbiprofen, and 6 mg tropisetron) were diluted with saline to make 200 mL. The background infusion rate was 2 mL/h, the patient controlled analgesic dose was 2 mL, and the locking time was 15 min. After the surgery, a visual analog scale scoring system was used to evaluate the degree of pain (0 points: painless, 10 points: severe pain) in order to maintain a visual analog scale score of 3 points or less.

### Determination of cognitive function

The same researcher who was blind to the grouping of the patients performed the MoCA (Beijing edition) to assess the patients’ neuropsychological statuses at the following time points: 3 days before surgery (T0) and Day 1 (T1), Day 2 (T2), Day 3 (T3), and Day 7 (T4) after the surgery. The MoCA assesses seven cognitive areas: visuospatial/execution, naming, attention, language, abstract, delayed recall, and orientation. The range of the total score is 0–30 points with 26 points or more indicating normal cognition. The test time was about 10 min.

### Collection and testing of blood samples

Peripheral blood samples (3 mL) were collected from each patient at each time point. The samples were centrifuged, and the plasma was preserved at −80 °C until further testing. Double-antibody sandwich enzyme-linked immunosorbent assay reagent kits (Shanghai Bogu Biotechnology Co., Ltd., Shanghai, China) were used to detect the serum levels of ADP and MMP-9.

### Statistical analysis

SPSS (version 17.0, IBM Corporation, Armonk, NY, USA) software was used for the analysis. The measurement data were expressed as mean ± standard deviation. Intragroup comparisons were assessed with repeated measure analyses of variance. The intergroup comparisons were made with *t* tests. The measurement data were assessed with *χ* ^2^ tests, and the relationships between the serum levels of ADP and MMP-9 and the MoCA scores in POCD patients were analyzed with linear correlations. *P* values less than 0.05 were considered statistically significant.

## Results

### General characteristics of the two groups

Age, body mass index, anesthesia time, operation time, intraoperative blood loss, and other variables did not differ significantly between the two groups (*P* > 0.05, Table 1).

**Table 1:** Comparison of general information between the two groups ($\bar{x}$ ± s)

Group	*n*	Age	BMI	Anesthesia time (min)	Operation time (min)	Intraoperative blood loss (mL)
Non-POCD group	28	75.33 ± 3.30	22.63 ± 2.33	101.63 ± 16.56	83.63 ± 12.33	346.38 ± 73.63
POCD group	70	75.13 ± 5.60	22.38 ± 3.03	102.13 ± 17.63	82.39 ± 17.53	348.39 ± 78.38

### MoCA scores of the two groups

The MoCA scores did not differ significantly between the two groups at T0 (*P* > 0.05). The MoCA scores of the POCD group at T1, T2, T3, and T4 were significantly lower than that at T0, while those of the non-POCD group at T1 and T2 were significantly lower than that at T0, and restored to that at T0 from T3. The intergroup comparison showed that the MoCA scores of the POCD group at each time point were significantly lower than those of the non-POCD group (*P* < 0.05, Table 2).

**Table 2:** Comparison of MoCA between the two groups at different time points ($\bar{x}$ ± s)

Group	*n*	T0	T1	T2	T3	T4
Non-POCD	28	28.63 ± 1.63	25.86 ± 1.39^a^	26.73 ± 1.61^a^	28.18 ± 1.31	28.31 ± 1.33
POCD	70	28.83 ± 1.33	22.53 ± 1.23^ab^	23.36 ± 1.67^ab^	24.33 ± 1.84^ab^	25.98 ± 1.16^ab^

Compared with T0, ^a^ *P* < 0.05; compared with the non-POCD group, ^b^ *P* < 0.05

### Serum levels of ADP and MMP-9 in the two groups

In the POCD group, the MMP-9 serum levels at T1, T2, T3, and T4 were significantly higher than the levels at T0 (*P* < 0.05), while the ADP serum levels at T1, T2, T3, and T4 were significantly lower than the levels at T0 (*P* < 0.05). In the non-POCD group, the MMP-9 serum levels at T1 and T2 were significantly increased compared to the T0 level (*P* < 0.05), while the ADP serum levels at T1 and T2 were significantly decreased compared to the T0 levels (*P* < 0.05), and the levels of both were restored to the preoperative level at T3. The intergroup comparison showed that the serum MMP-9 levels of the POCD group at each postsurgical time point were significantly higher than those of the non-POCD group (*P* < 0.05), and the serum ADP levels of the POCD group at each postsurgical time point were significantly lower than those of the non-POCD group (*P* < 0.05, Table 3).

**Table 3:** Comparison of serum levels of ADP and MMP-9 between the two groups at different time points ($\bar{x}$ ± s)

Time point	MMP-9 (μg/L)		ADP (mg/L)	
	Non-POCD group	POCD group	Non-POCD group	POCD group
*n*	70	28	70	28
T0	297.9 ± 47.3	293.3 ± 49.8	9.43 ± 1.32	9.39 ± 1.36
T1	438.6 ± 53.3^a^	536.6 ± 67.6^ab^	5.73 ± 1.03^a^	4.33 ± 1.61^ab^
T2	386.6 ± 66.3^a^	481.2 ± 63.7^ab^	6.66 ± 1.38^a^	4.66 ± 1.23^ab^
T3	301.3 ± 67.3	431.9 ± 63.9^ab^	9.13 ± 1.03	5.13 ± 1.63^ab^
T4	296.9 ± 53.6	393.1 ± 48.3^ab^	9.38 ± 1.17	6.03 ± 1.13^ab^

Compared with T0, ^a^ *P* < 0.05; compared with the non-POCD group, ^b^ *P* < 0.01

### Relationships of serum ADP and MMP-9 levels with the MoCA score in the POCD group

The linear correlation analysis showed that the serum MMP-9 levels were significantly and negatively correlated with the MoCA score (*r* = −0.833, *P* < 0.01), while the serum ADP levels were significantly and positively correlated with the MoCA score (*r* = 0.513, *P* < 0.01).

## Discussion

POCD is a common postoperative complication in elderly patients, and the incidence in elderly patients after noncardiac surgery ranges from 13 to 47 % [11]. This study showed that the incidence of POCD in elderly patients was 28.5 %, which was consistent with the findings of previous reports. The pathogenesis of POCD is complex. Anesthesia, hypoxemia, hypotension, surgical type, anesthetic type, sleep disorders, and inflammatory reactions all contribute to POCD [12], but the exact mechanism is still unclear. Currently, there are no uniform standards for the diagnosis of POCD, and its diagnosis is still mainly based on neural psychological measurements. The most commonly used tools in recent years are the Mini-Mental State Examination (MMSE) and MoCA. However, the MMSE has a ceiling effect, and it is therefore not sensitive enough to detect mild POCD cases. In addition, it is greatly affected by education, and higher education can produce false negative results [13]. The MoCA scoring system, which is suitable for wide age ranges and educational levels, exhibits higher sensitivity and specificity than the MMSE [14]. The MoCA scoring system that was used in this study was the adapted Beijing version. MoCA scores less than 26 points indicate the presence of POCD. This study examined elderly patients who required total hip replacement surgery, experienced similar surgical trauma, and had a high incidence rate of POCD. However, preoperative anxiety, which is common and which would be much more obvious close to the time of the surgery, might have affected the earlier preoperative assessment and reduced the impact of negative emotions on the assessment results. Therefore, we performed the MoCA assessment 3 days before the surgery and strictly controlled the patient’s preoperative cognitive function state and the amount of anesthetics during the surgery. Factors that might affect postoperative cognitive function, including hypoxemia and hypotension, were avoided in order to obtain comparable results.

Adiponectin is abundant in human plasma, and its expression and secretion are regulated by a number of inflammatory cytokines, reactive oxygen species, transcription factors, and hormones, such as tumor necrosis factor-α and interleukin-6, and other inflammatory mediators could inhibit the expression and secretion of ADP. ADP has a wide range of cardiovascular effects, including endothelial function improvements, myocardial hypertrophy remodeling inhibition, and anti-apoptosis and anti-inflammatory effects. Recently, the expression of ADP and its receptor (AdipoR) was observed throughout the pituitary gland and brain. AdipoR1 expression in the hypothalamus and basal ganglia suggests that ADP might be involved in the signaling pathway that controls energy balance as well as certain higher brain functions [15]. Kamogawa et al. [16] found that the increased serum levels of ADP in males had protective effects towards the progression of mild cognitive dysfunction. Studies have suggested that ADP might improve insulin resistance, thus improving blood–brain barrier functions and brain receptor functions [17]. Sakr [18] found that increasing the expression of AdipoR1 improved cognitive functions and the levels of acetylcholine in the hypothalamus of diabetic rats. The present study found that serum ADP levels at T1, T2, T3, and T4 in the POCD group were significantly lower than that at T0, and the serum ADP levels of the POCD group at each postsurgical time point were significantly lower than those of the non-POCD group. These results indicated that the higher incidence of POCD in elderly patients in the early post-general anesthesia stage might be related to a reduction in serum ADP levels and that ADP might delay the development of POCD to some extent. Further statistical analyses showed that the serum ADP levels were significantly and positively correlated with the MoCA scores, which suggested that ADP was a protective factor for POCD and that its effects might be achieved through an anti-inflammatory mechanism.

In recent years, a number of studies have found close relationships between MMP-9 and cerebrovascular diseases, and these relationships were much more obvious in the pathological processes underlying inflammatory responses, cerebral edema, and secondary brain injuries after the onset of cerebral infarction. These processes affect the degradation balance of the extracellular matrix, resulting in the occurrence of several pathological processes, such as blood–brain barrier damage and demyelination. MMP-9 is expressed at low levels in normal brain tissues. Inflammatory cytokines and hypoxic factors and nerve cells, microglial cells, and vascular endothelial cells synthesize MMP-9 and degrade the extracellular matrix, thus aggravating inflammation and increasing the permeability of the blood–brain barrier, brain damage, and neurological damage. Serum MMP-9 levels have been shown to be positively correlated with the severity of cognitive impairment [19]. Gaudet reported that POCD in elderly patients after cardiac surgery was related to increased MMP-9 levels [20]. The present study showed that the serum MMP-9 levels at T1, T2, T3, and T4 in the POCD group were significantly higher than the levels at T0, while the levels in the non-POCD group returned to preoperative levels at T3. Further statistical analysis showed that the serum levels of MMP-9 in the POCD group were significantly and negatively correlated with the MoCA scores, which indicated that the higher the postoperative serum MMP-9 levels were, the lower the MoCA score was. These findings suggested that the increased serum MMP-9 levels in elderly patients after general anesthesia might be involved in the pathophysiological process of POCD. In addition, we found that the MoCA scores of 11 patients in the POCD group restored normal 14 days after the surgery, the serum ADP was significantly increased, and the serum MMP-9 was significantly decreased. The post-discharge follow-up revealed that the MoCA scores of 30 patients restored normal 30 days after the surgery, and the serum ADP and MMP-9 restored normal; while the MoCA scores of the other four patients restored normal 90 days after the surgery.

In summary, the findings of this study suggested that the decreased serum ADP levels and increased serum MMP-9 levels in elderly patients after general anesthesia might be involved in the pathophysiological process of POCD.
